# *PRDM* Histone Methyltransferase mRNA Levels Increase in Response to Curative Hormone Treatment for Cryptorchidism-Dependent Male Infertility

**DOI:** 10.3390/genes9080391

**Published:** 2018-08-01

**Authors:** Faruk Hadziselimovic, Gieri Cathomas, Gilvydas Verkauskas, Darius Dasevicius, Michael B. Stadler

**Affiliations:** 1 Cryptorchidism Research Institute, Kindermedizinisches Zentrum Liestal, Bahnhofplatz 11, CH-4410 Liestal, Switzerland; 2 Department of Pathology Kantonspital Liestal, 4410 Liestal, Switzerland; gieri.cathomas@ksbl.ch; 3 Children’s Surgery Centre, Faculty of Medicine, University of Vilnius, 01513 Vilnius, Lithuania; gilvydas.verkauskas@vuvl.lt; 4 Institute for Pathology, National Centre of Pathology, Affiliate of Vilnius University Hospital Santariskiu Klinikos, 08406 Vilnius, Lithuania; darius.dasevicius@vpc.lt; 5 Swiss Institute of Bioinformatics, 4058 Basel, Switzerland; michael.stadler@fmi.ch; 6 Friedrich Miescher Institute for Biomedical Research, 4058 Basel, Switzerland

**Keywords:** PRDM, cryptorchidism, GnRHa, infertility, RNA-sequencing

## Abstract

There is a correlation between cryptorchidism and an increased risk of testicular cancer and infertility. During orchidopexy, testicular biopsies are performed to confirm the presence of type A dark (Ad) spermatogonia, which are a marker for low infertility risk (LIR). The Ad spermatogonia are absent in high infertility risk (HIR) patients, who are treated with a gonadotropin-releasing hormone agonist (GnRHa) to significantly lower the risk of infertility. Despite its prevalence, little is known about the molecular events involved in cryptorchidism. Previously, we compared the transcriptomes of LIR versus HIR patients treated with and without hormones. Here, we interpreted data regarding members of the positive regulatory domain-containing (*PRDM*) family; some of which encoded histone methyltransferases that are important for reproduction. We found there were lower levels of *PRDM1*, *PRDM6*, *PRDM9*, *PRDM13*, and *PRDM14* mRNA in the testes of HIR patients compared with LIR patients, and that *PRDM7, PRDM9*, *PRDM12*, and *PRDM16* were significantly induced after GnRHa treatment. Furthermore, we observed *PRDM9* protein staining in the cytoplasm of germ cells in the testes from LIR and HIR patients, indicating that the mRNA and protein levels corresponded. This result indicated that the curative hormonal therapy for cryptorchidism involved conserved chromatin modification enzymes.

## 1. Introduction

Cryptorchidism confers an elevated risk of testicular cancer and infertility and has been the focus of several studies [1,2,3]. While genetic risk factors have been identified, the etiology of cryptorchidism and the best clinical treatment remain a matter of debate [2,4]. Importantly, the roles of epigenetic alterations in cryptorchidism are not completely understood. We demonstrated previously that the presence of type A dark (Ad) spermatogonia in the testis, was a marker for low infertility risk (LIR); whilst their absence or low levels (below a critical threshold) indicated high infertility risk (HIR) [5,6]. Importantly, sustained treatment with a gonadotropin-releasing hormone agonist (GnRHa, Buserelin) enabled the Ad spermatogonia population to recover; thus, it significantly improved fertility in HIR patients [7].

One major goal of work in this field is to elucidate the molecular mechanisms that underlie cryptorchidism. To this end, well-established genetic and genomic approaches have been used to identify proteins and long non-coding RNAs that may be relevant to this disease [8,9,10,11,12,13,14], reviewed in [15]. More recently, epigenetic processes have shown promise to better understand the etiology of cryptorchidism. Epigenetics includes a wide variety of modifications of DNA (typically cytosine methylation); histones, which form chromatin (for example, methylation and acetylation); and more recently, RNA [16,17,18,19].

Ongoing studies in our laboratory have analyzed and compared the transcriptomes of LIR patients, HIR patients, HIR patients that received GnRHa treatment, and untreated controls [8,9]. These studies were performed using testicular biopsies and DNA strand-specific RNA sequencing based on the Illumina system. This work confirmed and vastly extended earlier studies that were based on Gene Chip technology [10] and identified genes that, were both differentially expressed in LIR/HIR samples and showed altered mRNA levels in response to GnRHa treatment. 

The positive regulatory domain-containing (*PRDM*) family members, have an N-terminal positive regulatory (PR) domain that is structurally related to the SET domain methyltransferase domain, as well as a variable number of zinc fingers that are required for protein−DNA and protein−protein interactions. The PRDM genes are important in disease [20] and for diverse developmental processes [21], including primordial germ cell specification and differentiation (*PRDM1/BLIMP1* [22], *PRDM14* [23]; reviewed in Reference [4]), embryo development (*PRDM12*, see IMPC [24]), and adult germ cell meiotic r24ecombination (*PRDM9* [25]). In this study, we focused on the transcriptional response of the *PRDM* genes in LIR and HIR patients, versus HIR patients treated with GnRHa. We found that, five *PRDM* genes were down-regulated in HIR patients and four were induced after GnRHa treatment. Among them was *PRDM9*, which is essential for germ cell development and fertility. *PRDM9* contains three characteristic *PRDM* domains: A Krüppel-associated box zinc finger (KRAB-ZFPs) domain implicated in protein−protein interactions; a PR/SET domain with histone methyltransferase activity; and a zinc finger domain for DNA recognition and binding [26]. The *PRDM9*-bound hot spot DNA is brought to the chromosomal axis by interaction with other proteins, serving as a link between *PRDM9* and the cohesin/synaptonemal complex proteins, thereby assuring a proper spatial environment for double strand breaks initiation and repair [26]. It is known that in the absence of *PRDM9*, double strand breaks are mainly formed at promoters where they cannot be repaired properly, which leads to germ cell apoptosis [27]. Of importance, *PRDM9* was significantly induced by GnRHa treatment. This result implicates chromatin modification enzymes in cryptorchidism and curative hormone treatment.

## 2. Materials and Methods

### 2.1. Study Population and Biopsy Sample Collection

Testis localized outside of the scrotum and incapable of being brought into a stable scrotal position, is defined as a cryptorchid testis. In our earlier studies, all patients with isolated cryptorchidism had undescended testes located in the inguinal region [9,28]. Patients were age and ethnicity matched. The age of the patients ranged from 8 to 59 months, resulting in a median age of 18.5 months. Testicular biopsies were taken at the time of orchidopexy. Collected biopsy samples were divided into two pieces, with one fragment immediately immersed in RNAlater (ThermoFisher Scientific, Waltham, MA, USA) and stored at −25 °C until further processing (for RNA extraction and RNA-sequencing), and the other fixed in glutaraldehyde for histological processing. To evaluate gene expression profiles, we used RNA sequencing data from our two previous studies. The first study included 15 biopsies of 15 patients (7 unilateral and 8 bilateral undescended testes), which were selected prior to randomization and based on histological results. Seven patients were grouped into the High Infertility Risk group lacking Ad spermatogonia (HIR/Ad−), and 8 patients were grouped into the Low Infertility Risk group presenting Ad spermatogonia (LIR/Ad+) [9,28,29]. From randomized study, in which Ad-bilateral cryptorchid boys were treated with GnRHa (Buserelin) after the first orchidopexy (surgery), data was retrieved from 4 patients. Initial biopsies of these four patients revealed no Ad spermatogonia, indicating defective mini-puberty (Ad-group) [29]. The second testis was managed by orchidopexy and biopsied 6 months after the initial surgery and GnRHa treatment [28,29]. Since data of first biopsies of two out of these four patients was retrieved from the HIR(Ad−)/LIR(Ad+) comparison study (15 biopsies), in total results from 21 biopsies were compared. 

### 2.2. Immunohistochemical Analyses

Immunohistochemistry experiments were performed with four biopsies comprising, one control adult testis with complete spermatogenesis obtained post-mortem; one from a LIR patient; and two from HIR patients. Biopsies from cryptorchid testes were split in two. One part was embedded in EPON and fixed in glutaraldehyde for exact determination of the number of Ad spermatogonia. To visualize the histology of testicular cells, the samples were counterstained with toluidine blue. For immunohistochemical analysis, the second part of the testicular tissue was fixed in paraformaldehyde. The sections were treated with 2% bovine serum albumin to reduce non-specific binding, and then incubated with primary antibody overnight at 4 °C. All samples were washed with PBS between incubations. We used an anti-*PRDM9* polyclonal antibody (Sigma-Aldrich (St. Louis, MO, USA) HPA059555, dilution 1:25 with Bond Primary Antibody Diluent (AR6352), and pretreatment EDTA-puffer AR9640 for 30 min., 95 °C). The secondary antibodies were conjugated with alkaline phosphatase (Bond Polymer Refine Red Detection DS9390) and were used to detect binding of the primary antibody. The chromogenic reaction was performed with fast red as per kit (DS9390). The Fast-Red reaction was terminated by washing with Bond Wash-puffer (AR9590, pH 7.6). Antibody binding was indicated by a red precipitate. Different cell types were identified based on their nuclear morphology and position within the developing gonad. Controls for non-specific binding of the secondary antibody were performed by omitting the primary antibody; these consistently yielded no signal within the seminiferous epithelium or the interstitial space. The experimental design, biomaterials and treatments, reporters, staining, imaging data, and image characterization were performed in compliance with the minimum information specification for immunohistochemistry experiments.

### 2.3. RNA Preparation, Sequencing, Data Analyses, and RNA Expression Levels

The workflow from RNA isolation, through to purification, library preparation, sequencing, data analyses, and expression level analysis, was described earlier in detail in References [9,28].

Data and Differential Gene Expression Analyses Determination of differentially expressed genes, statistical analyses and model design were described previously [9,28]. Only genes with at least one read per million, in at least two samples, were included. *p* values and fold-changes, were calculated for the treatment factor, and differentially expressed genes were defined as those displaying a false discovery rate (FDR) of less than 0.05. Raw data files were deposited at the Database of Genotypes and Phenotypes (dbGaP) with the accession number phs001275.v1.p1.

### 2.4. RNA-Sequencing Data and Differential Gene Expression Analysis

The procedures for generating RNA-Sequencing (RNA-Seq) data, identifying differentially expressed genes were described previously [9,28]. Briefly, we identified genes with at least one read per million in at least two samples. The *p*-values and fold-changes in expression were calculated for the treatment factor, and differentially expressed genes were selected with a false discovery rate (FDR) <0.05 and ≥2-fold changes in expression levels. 

### 2.5. Ethics Statement

Investigations were carried out in accordance with the Declaration of Helsinki of 1975 (revised in 2008). The study was approved by the Institutional Review Board and the Independent Ethics Committee of Vilnius University (Vilnius Regional Biomedical Research Ethics Committee, No. 158200-580-PPI-17, 11 June 2013). Written informed consent was obtained from the patients’ guardians after approval by the ethical committee.

## 3. Results

The *PRDM* family comprises 16 members that are consecutively numbered. The current nomenclature used by the human proteome reference annotation database neXtProt (www.nextprot.org) for *PRDM3* is *MECOM* [30]. The following five PRDM genes had lower mRNA levels in HIR patients than in LIR patients: *PRDM1* is important for the immune system and germ cell development (logFC = −1.11, *p* = 2.1 × 10^−4^, FDR = 0.003); *PRDM6* for smooth muscle development (logFC = −2.15, *p* = 1.1 × 10^−6^, FDR = 0.0002); *PRDM9* for meiotic recombination (logFC = −1.17, *p* = 6.3 × 10^−4^, FDR = 0.007); *PRDM13* (logFC = −1.58, *p* = 3.6 × 10^−3^, FDR = 0.024) is a novel crucial component of the Ptf1a regulatory pathway that, by modulating the transcriptional activity of basic helix−loop−helix factors, such as Neurog2, controls the balance between GABAergic and glutamatergic neuronal fate in the dorsal and caudal part of the vertebrate neural tube [31]; and *PRDM14* is involved in stem cell/germ cell differentiation (logFC = −2.55, *p* = 5.2 × 10^−4^, FDR = 0.001) [30] (Table 1). 

**Figure 1:**
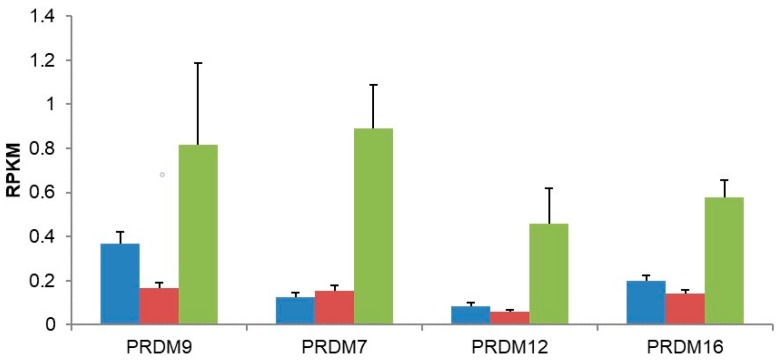
Increases in *PRDM* gene expression following GnRHa treatment. Results are presented as reads per kilobase and per million (RPKM), and the median and median absolute deviation (MAD) values are presented. Blue bars represent low infertility risk (LIR) untreated testes, red bars represent high infertility risk (HIR) testes before treatment, and green bars represent HIR testes after GnRHa treatment.

Importantly, *PRDM9* was induced in HIR patients following treatment with gonadotropin-releasing hormone agonist (GnRHa) (logFC = 1.68, *p* = 2.2 × 10^−4^, FDR = 0.0014), (Table 2, Figure 1). Moreover, three other *PRDM* genes did not differ between untreated LIR and HIR samples but were upregulated after treatment: *PRDM7* (logFC = 2.18, *p* = 2.16 × 10^−5^, FDR = 0.0003); *PRDM12* (logFC = 2.11, *p* = 3.38 × 10^−3^, FDR = 0.0113); and *PRDM16* (logFC = 1.15, *p* = 1.1 × 10^−3^, FDR = 0.0044). Finally, *PRDM4* (logFC = −0.53, *p* = 7.5 × 10^−3^, FDR = 0.0195) and *PRDM5* (logFC = −0.53, *p* = 10^−2^, FDR = 0.0248) were downregulated, (Table 2, Figure 1).

**Figure 2:**
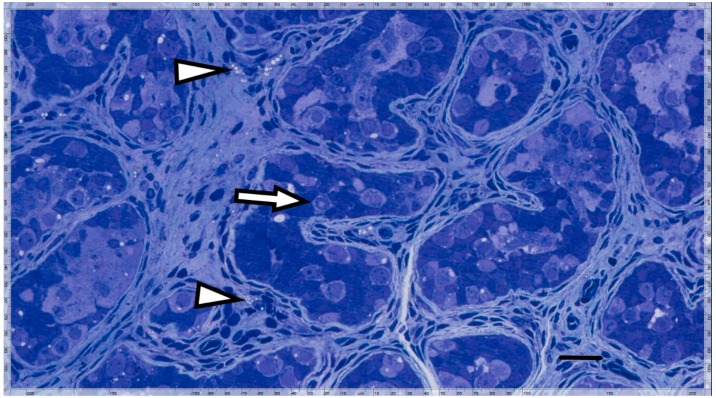
Semi-thin section from a LIR patient’s testis displaying precise testicular structure and Ad spermatogonia with a nuclear rarefaction zone (arrow). Juvenile Leydig cells are atrophic (arrowhead) (horizontal bar; 30 µ).

**Figure 3:**
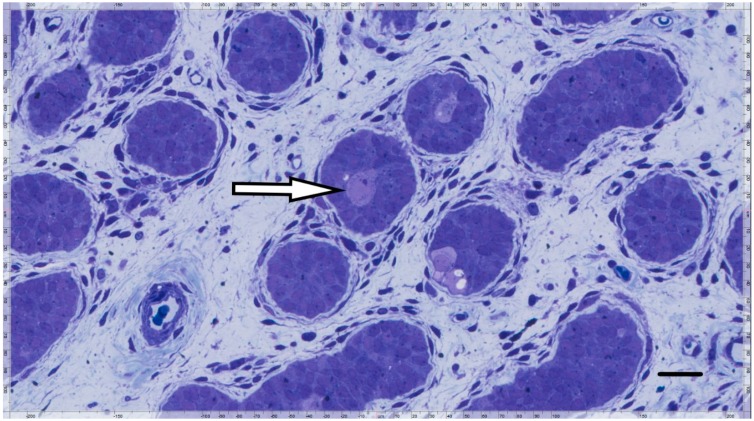
Semi-thin section from a HIR patient’s testis with no Ad spermatogonia and an untransformed gonocyte (arrow). The broad, fibrotic interstitial space harbors atrophic juvenile Leydig cells (horizontal bar; 30 µ).

Other than *PRDM9*, none of the GnRHa responsive genes are known to be important for germline development [30]. We note that on its website, the International Mouse Phenotyping Consortium (IMPC, www.mousephenotype.org) reported somatic effects but no fertility defects in mouse lines where *Prdm4* and *Prdm12* are mutated. In contrast, male and female mice lacking *Prdm14* were infertile [32]. The LIR patient’s testicular biopsy showed complete prepubertal spermatogenesis, Ad spermatogonia, and total germ cell count of 1 per tubular cross section (Figure 2); while, testes from the HIR patients showed no Ad spermatogonia, and an average of 0.19 and 0.17 germ cells per tubule (Figure 3).

**Figure 4:**
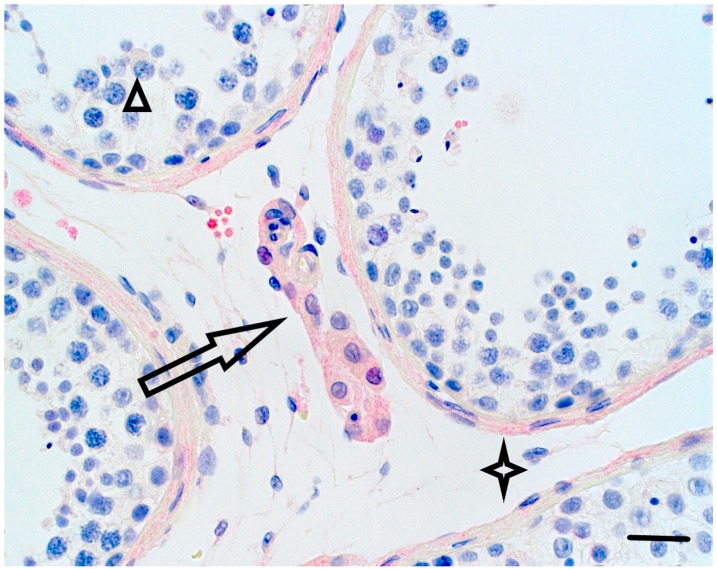
The control adult testis displayed complete spermatogenesis. PRDM9 protein (red) staining in the control adult testis with complete spermatogenesis. The large arrow is pointing at Leydig cells showing medium strong PRDM9 staining; while, the arrow head is indicating spermatocytes with weak cytoplasmic staining. The star points to positively stained peritubular connective tissue. Of note, PRDM9 was mainly localized in the cytoplasm (Horizontal bar; 30 µ).

**Figure 5:**
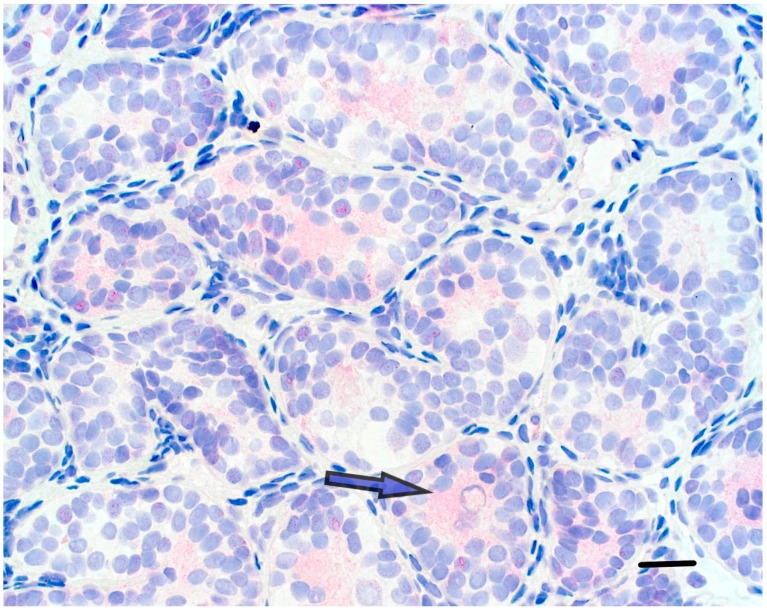
A testis from a HIR patient displaying weak PRDM9 cytoplasmic staining of the germ cells (arrow). Horizontal bar; 30 µ.

We found positive weak−to−moderate staining for PRDM9 in the cytoplasm of spermatogonia in the control, as well as in the testes from inHIR patients (Figure 4 and Figure 5).

**Figure 6:**
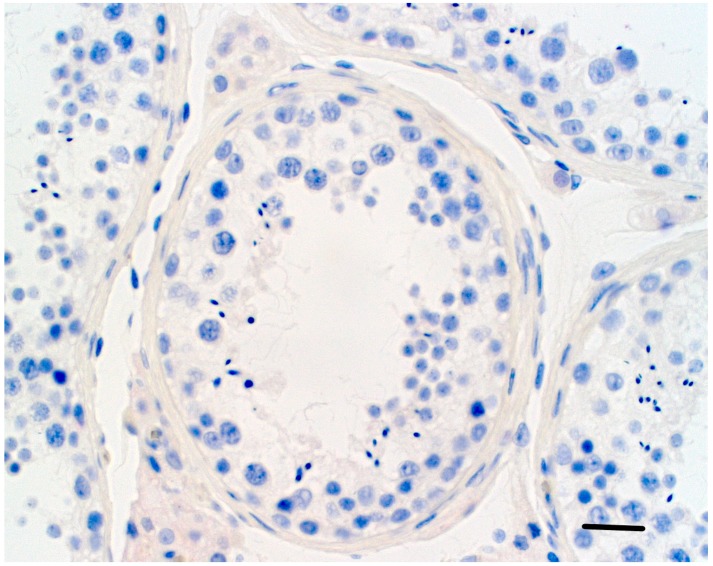
A control adult testis without PRDM9 antibodies, lacking red protein staining. (Horizontal bar; 30 µ).

Furthermore, there was moderate positive staining for PRDM9 in the control testis in the Leydig cell cytoplasm, nucleus and peritubular connective tissue (Figure 4). No red staining was observed in antibody negative control testis (Figure 6).

## 4. Discussion

When interpreting testicular RNA-Seq data, it is of interest to focus on enzymes that are involved in epigenetic processes. Notably, changes at the mRNA level do not necessarily mean changes at the protein level. However, fluctuating RNA levels, determined by RNA profiling, are a good indication that a given gene is implicated in a process. Although qualitative immunohistochemistry is not suitable for detection of subtle differences between HIR and LIR patients, it still suggests that the protein is present in spermatogonia. For the first time, we reported positive PRDM9 protein staining in the cytoplasm of the germ cells in a prepubertal testis. We speculate that PRDM9 may play a novel role in the cytoplasm of spermatogonia. 

This work focused on 16 members of the *PRDM* family of histone demethylases in the context of curative hormonal treatment of cryptorchidism-dependent infertility. *PRDM1* and *PRDM14* mutants cannot form primordial germ cells [33,34,35]. Both genes coordinate somatic epiblast gene expression during primordial cell specification and promote primordial germ cell pluripotency [36,37]. Specification also involves genome-wide epigenetic reprograming [37]. Loss of *PRDM14* leads to drastic shrinkage and even disappearance of the hypomethylated domains in epiblast cells, but not the demethylation-sensitive domains in primordial germ cell-like cells [38]. *PRDM12* is induced by retinoic acid, a key hormone for meiotic development, and it is required for sensory neuron development and pain perception [39,40,41,42]. In mice, the gene is required for normal embryogenesis and it fulfils an essential role during post-natal development (IMPC at www.mousephenotype.org; [32]). Studies in *Prok2* receptor mutant mice and *Prok2*-/-mice showed olfactory bulb defects and disrupted GnRH neuron migration, resulting in a dramatic decrease in the GnRH neuron population in the hypothalamus as well as hypogonadotropic hypogonadism development [43,44]. Our RNA profiling data strongly supported the theory that, in the HIR group of cryptorchid boys, insufficient *PROK2* gene expression induces deficient luteinizing hormone secretion, resulting in impaired mini-puberty and infertility [9]. Of interest, *PRDM13* is implicated in olfactory placode development [45]. Furthermore, the *NKX2*-homeobox gene is a stimulator of *PRDM13* [44,45]; and *NKX2-3* (log FC = −3.36, FDR = 0.0073) and *NKX2-4* (log FC = −2.54, FDR = 0.004) were downregulated in the HIR group. Following GnRHa treatment, *NKX2-2* showed increased expression (log FC = 2.51, FDR = 0.0064), indicating a possible role for this homeobox gene in cryptorchidism-induced infertility. *PRDM16* is important for neural differentiation and *PRDM7* is a Histone 3 Lysine 4 Tri-methyltransferase, but no role has been reported yet for this protein [46,47,48]. Interestingly, the PR domain-containing protein 7 is the result of a recent gene duplication of *PRDM9* [49]. The GnRHa treatment increased PRDM7 gene expression (Figure 1). Given the four upregulated *PRDM* genes (*PRDM* 7/9/12/16), there may be a link between PRDM14 function and PRDM16, where PRDM14 mediates the acquisition of germ cell pluripotency, in part by upregulating SOX2 [37].

*PRDM9* was down-regulated in HIR patient samples and responded positively to GnRHa treatment. This observation was intriguing in light of *PRDM9*’s critical role in establishing meiotic recombination in the male germline [49]. Altered expression signals in biopsies from undescended testes suggested that germ cells were affected, since the major function of PRDM9 is in meiotic spermatocytes. Furthermore, in the absence of *PRDM9*, double-strand breaks are formed at other available H3K4me sites mainly located in promoters; these cannot be repaired properly, and germ cells undergo apoptosis [27]. However, it is also possible that other testicular cell types respond to the birth defect, since in mice *Prdm9* is expressed in Sertoli cells and in the entire germline. 

In our young patients, spermatogenesis is not yet established, and meiotic recombination has not yet occurred, so our main result suggests that the *PRDM9* protein has a novel role in dividing mitotic spermatogonia as well as in inducing Ad spermatogonia development. This is in accordance with a recent report that implicates *PRDM9* in events other than recombination, such as gene activation in cultured cells [50]. Thus, *PRDM9* gene product is a downstream effector of testosterone action and is related to testosterone-regulated cell proliferation and differentiation in classical testosterone target tissues. Of note, *VCX* (logFC = −3.2, *p* = 1.76 × 10^−7^, FDR = 0.00015) and *CTCFL* (logFC = −0.7, *p* = 0.002, FDR = 0.01), which are known *PRDM9* target genes, were also down-regulated in HIR patients. Both spermatogenesis specific genes are important for germ cell proliferation, apoptosis, and ribosome assembly [51,52]. Furthermore, *CTCFL* is a male germ cell gene regulator [53]. However, no increase in gene expression occurred following GnRHa treatment, indicating that these two genes had limited importance in the transition from gonocyte to Ad spermatogonia. (*CTCFL*; logFC = −0.33, *p* = 0.11, FDR = 0.1, *VCX*; logFC *=* 0.10, *p* = 0.78, FDR = 0.83).

In conclusion, we found that the mRNA levels of five *PRDM* genes were lower in the testes from HIR patients compared with LIR patients, and that four *PRDM* genes were significantly induced after GnRHa treatment. As central players in development, *PRDM* genes act at different levels in the hypothalamus-pituitary-testicular axis. *PRDM13* acts at the hypothalamic level; while, *PRDM9* is directly implicated in the gonocyte to Ad spermatogonia transition and fertility development. 

## Figures and Tables

**Table 1:** Log-transformed fold-changes (logFC) in the expression of positive regulatory domain-containing (*PDRM)* genes between low infertility risk and high infertility risk patients.

ENTREZID	SYMBOL	IogFC HIR/LIR	FDR HIR/LIR	Pvalue HIR/LIR
639	PRDM1	−1.11	0.003	0.000217
7799	PRDM2	0.29	0.059	0.012406
2122	MECOM	−0.36	0.12	0.028340
11108	PRDM4	0.07	0.46	0.289401
11107	PRDM5	−0.23	0.244	0.107015
93166	PRDM6	−2.15	0.0002	1.1 × 10−6
56978	PRDM8	−0.13	0.807	0.693591
56979	PRDM9	−1.17	0.007	0.000639
56980	PRDM10	−0.12	0.255	0.114300
56981	PRDM11	0.25	0.109	0.031354
59336	PRDM13	−1.58	0.024	0.003636
63978	PRDM14	−2.55	0.001	5.2 × 10−5
63977	PRDM15	−0.25	0.103	0.103668
63976	PRDM16	−0.42	0.225	0.094767

**Table 2:** Log-transformed fold-changes (logFC) in the expression of the indicated genes in high infertility risk patients before and after gonadotropin-releasing hormone agonist treatment.

ENTREZID	SYMBOL	IogFC HIR/LIR	FDR HIR/LIR	Pvalue HIR/LIR
11108	PRDM4	−0.53	0.0105	0.007595
11107	PRDM5	−0.53	0.0248	0.010239
11105	PRDM7	2.18	0.0003	2.1 × 10^−5^
56979	PRDM9	1.68	0.0014	0.000219
59335	PRDM12	2.11	0.0113	0.031354
63976	PRDM16	1.15	0.0044	0.001111
